# 接受CAR-T细胞治疗的复发/难治性B细胞非霍奇金淋巴瘤患者新型冠状病毒感染特点及其影响因素

**DOI:** 10.3760/cma.j.issn.0253-2727.2023.10.006

**Published:** 2023-10

**Authors:** 童 葛, 辉 刘, 镇灏 王, 阳 曹, 义成 张, 亮 黄, 文斌 钱, 晓曦 周

**Affiliations:** 1 华中科技大学同济医学院附属同济医院血液内科，武汉 430030 Department of Hematology, Tongji Hospital, Tongji Medical College, Huazhong University of Science and Technology, Wuhan 430030, China; 2 浙江大学医学院附属第二医院血液内科，杭州 310009 Department of Hematology, the Second Affiliated Hospital, College of Medicine, Zhejiang University, Hangzhou 310009, China

**Keywords:** B细胞非霍奇金淋巴瘤, 嵌合抗原受体T细胞, 新型冠状病毒, 冠状病毒感染, B-cell non-Hodgkin lymphoma, Chimeric antigen receptor T-cells, COVID-19, COVID-19 infection

## Abstract

**目的:**

探究复发/难治性B细胞非霍奇金淋巴瘤（R/R B-NHL）患者接受嵌合抗原受体T细胞（CAR-T细胞）治疗前后感染新型冠状病毒（新冠病毒）的临床特点及治疗情况，以及接受CAR-T细胞治疗的患者新冠病毒重症感染的影响因素。

**方法:**

回顾性研究2017年12月至2023年2月于华中科技大学同济医学院附属同济医院血液内科和浙江大学医学院附属第二医院血液内科接受CAR-T细胞治疗，并且在2022年12月至2023年2月首次感染新冠病毒的59例R/R B-NHL患者的资料，根据新冠病毒感染严重程度将患者分为轻型、中型、重型、危重型，对各组临床特征进行组间比较，并采用单因素Logistic回归分析影响新冠病毒重症感染的影响因素。同时对B细胞再生障碍（BCA）及非BCA组病例的临床特征进行比较。

**结果:**

59例患者新冠病毒感染临床分型：39例（66.1％）轻型，9例（15.3％）中型，10例（16.9％）重型，1例（1.7％）危重型。年龄>55岁、接受过自体造血干细胞移植、诊断新冠病毒感染时为疾病进展状态以及BCA状态是新冠病毒重症感染的影响因素。BCA组患者新冠病毒感染严重程度较非BCA组更重（*P*<0.001），新冠病毒阳性持续时间更长（*P*＝0.015），抗病毒治疗周期更久（*P*<0.001），住院治疗率更高（*P*<0.001）。

**结论:**

在CAR-T细胞治疗的R/R B-NHL患者中，积极预防及治疗新冠病毒感染仍是亟须关注的问题。

## Abstract (en)

**Objective:**

To explore the clinical characteristics and treatment of COVID-19 infection in patients with relapsed/refractory B-cell non-Hodgkin lymphoma before and after receiving chimeric antigen receptor T-cell therapy, and study the influencing factors of severe COVID-19 infection in these patients.

**Methods:**

The data of 59 patients with relapsed/refractory B-cell non-Hodgkin lymphoma who received chimeric antigen receptor T-cell therapy at the Department of Hematology, Tongji Hospital, Tongji Medical College, Huazhong University of Science and Technology and Department of Hematology, the Second Affiliated Hospital, College of Medicine, Zhejiang University between December 2017 and February 2023, and who were infected with novel coronavirus between December 2022 and February 2023 were retrospectively studied. Patients were divided into light, medium, severe, and critical groups, and the differences between the groups were analyzed using the chi-square test. A univariate logistic regression model was used to evaluate the contribution of each variable and its relationship with severe infection. The chi-square and Fisher's exact tests were used to analyze the differences between the B-cell aplasia and B-cell recovery（BCR）groups.

**Results:**

Of the 59 pre- and post-infusion infections, 39（66.1％）led to mild COVID-19, 9（15.3％）resulted in moderate COVID-19, 10（16.9％）resulted in severe COVID-19, and 1（1.7％）led to critical COVID-19. Moroever, age greater than 55 years, having received autologous hematopoietic stem cell transplantation, progressive disease status, and B-cell aplasia at the time of diagnosis of COVID-19 infection are factors affecting severe infection. Patients with B-cell aplasia had a more severe infection with COVID-19（*P*<0.001）, a longer duration（*P*＝0.015）, a longer antiviral therapy course（*P*<0.001）, and a higher hospitalization rate（*P*<0.001）than the BCR group.

**Conclusion:**

Active prevention and treatment of COVID-19 infection remains a crucial issue requiring urgent attention in managing patients with relapsed/refractory B-cell non-Hodgkin lymphoma treated with chimeric antigen receptor T-cell therapy.

嵌合抗原受体T细胞（CAR-T细胞）治疗目前在复发/难治性B细胞非霍奇金淋巴瘤（R/R B-NHL）中已经取得显著疗效[1]–[2]。2021年6月和9月，国家药品监督管理局分别批准了阿基仑赛注射液和瑞基奥仑赛注射液上市，用于治疗既往接受二线或以上系统性治疗的成人R/R B-NHL。2019年12月起，新型冠状病毒（新冠病毒）感染在全球范围内蔓延[3]，血液病患者新冠病毒感染的临床特征及其影响因素受到广泛关注。既往报道显示，接受CAR-T细胞治疗的血液肿瘤患者新冠病毒感染的病死率为4.3％～41.4％[4]–[8]。R/R B-NHL患者免疫功能低下，接受CAR-T细胞治疗前的化疗和CAR-T细胞治疗可导致B细胞再生障碍（BCA）[9]，新冠病毒感染后重症率和病死率更高，并且更可能发生新冠病毒再感染或持续阳性。目前关于接受CAR-T细胞治疗患者新冠病毒感染临床特征及影响因素的报道仍然较少。本文通过回顾性分析，探讨接受CAR-T细胞治疗的R/R B-NHL患者新冠病毒感染情况及其影响因素。

## 病例与方法

1. 病例资料：本研究为回顾性队列研究，分析2017年12月至2023年2月于华中科技大学同济医学院附属同济医院血液内科和浙江大学医学院附属第二医院血液内科接受CAR-T细胞治疗、并在2022年12月至2023年2月首次感染新冠病毒的59例R/R B-NHL患者。患者均明确诊断为B-NHL且经过二线或以上系统性治疗后复发或疾病未缓解。按照新冠病毒感染严重程度[10]，将患者分为轻型、中型、重型和危重型，重型和危重型感染归为新冠病毒重症感染。新冠病毒再感染的定义参照文献[11]–[12]标准。

2. CAR-T治疗方案：CAR-T细胞静脉输注前5 d开始进行FC方案预处理：氟达拉滨25 mg·m^−2^·d^−1^，−5 d～−3 d，环磷酰胺250 mg·m^−2^·d^−1^，−5 d～−3 d。根据靶点对CAR-T细胞分类，CD19单靶点28例，其中阿基仑赛5例，瑞基奥仑赛13例，其他10例（ClinicalTrials，NCT02537977）；CD19/CD22双靶点10例（ClinicalTrials，NCT05091541）；CD19/CD20双靶点21例（ClinicalTrials，NCT04317885）。

3. 疗效评估及B淋巴细胞比例检测：根据2014年Lugano修订版的淋巴瘤疗效评价标准[13]对接受CAR-T治疗的患者进行疗效评估。采用抗人单克隆抗体CD45 V500c（美国BD公司产品）、CD19 PE-Cy7（美国Biolegend公司产品）检测患者外周血B淋巴细胞占淋巴细胞比例。BCA定义为CD19阳性淋巴细胞比例<3.0％[9]。

4. 统计学处理：采用GraphPad Prism 8.0和SPSS 22.0进行统计学分析。符合正态分布的连续变量以“*x±s*”表示，组间比较采用两独立样本*t*检验；不符合正态分布的连续变量采用中位数（范围）表示，组间比较采用两独立样本秩和检验。无序分类变量的组间比较采用卡方检验或Fisher精确概率法，有序分类变量的组间比较采用Mann-Whitney *U*检验（两组）或Kruskal-Wallis *H*检验（多组）。采用单因素Logistic回归分析新冠病毒重症感染的影响因素，四格表中存在零计数情况采用Haldane修正，即在每个单元格的计数中添加0.5，以避免在计算*OR*时出现除以零的错误。双侧*P*<0.05为差异有统计学意义。

## 结果

1. 基线特征：59例患者中位年龄为57（18～77）岁，年龄≥60岁的患者18例（30.5％），男性30例（50.8％）。疾病类型：弥漫大B细胞淋巴瘤50例（84.7％），滤泡性淋巴瘤4例（6.8％），原发纵隔大B细胞淋巴瘤3例（5.1％），套细胞淋巴瘤、灰区淋巴瘤各1例（1.7％）。16例（27.1％）患者合并基础疾病，包括心脑血管疾病（含高血压）、糖尿病、慢性阻塞性肺疾病、慢性肾功能不全、甲状腺癌。仅接受CAR-T治疗的患者42例（71.2％），接受过自体造血干细胞移植治疗的患者17例（28.8％）。根据新冠病毒感染严重程度分组，轻型39例（66.1％）、中型9例（15.3％），重型10例（16.9％），危重型1例（1.7％）。比较不同严重程度分组患者临床特征，结果显示既往接受自体造血干细胞移植治疗患者新冠病毒感染重型/危重型比例更高（*z*＝−2.798，*P*＝0.005），而不同年龄、性别、诊断、合并基础疾病、CAR-T细胞靶点分组与新冠病毒感染严重程度分组分布差异无统计学意义（表1）。

**表1:** 接受CAR-T细胞治疗R/R B-NHL患者基线特征及免疫功能与新冠病毒感染严重程度分组的差异性比较

特征	轻型（39例）	中型（9例）	重型/危重型（11例）	统计量	*P*值
年龄[例（%）]				−0.747	0.455
≥60岁	11（28.2）	2（22.2）	5（45.5）		
<60岁	28（71.8）	7（77.8）	6（54.5）		
性别[例（%）]				−1.919	0.055
男性	16（41.0）	7（77.8）	7（63.6）		
女性	23（59.0）	2（22.2）	4（36.4）		
诊断[例（%）]				−1.661	0.097
弥漫大B细胞淋巴瘤	31（79.5）	8（88.9）	11（100.0）		
其他^a^	8（20.5）	1（11.1）	0（0）		
合并基础疾病[例（%）]				4.617	0.099
无	30（76.9）	7（77.8）	6（54.5）		
1种	8（20.5）	0（0）	3（27.3）		
≥2种	1（2.6）	2（22.2）	2（18.2）		
CAR-T靶点[例（%）]				4.133	0.127
CD19	15（38.5）	5（55.6）	8（72.7）		
CD19/CD22	8（20.5）	0（0）	2（18.2）		
CD19/CD20	16（41.0）	4（44.4）	1（9.1）		
接受自体造血干细胞移植[例（%）]	7（17.9）	3（33.3）	7（63.6）	−2.798	0.005
感染新冠病毒时间[例（%）]				10.065	0.007
CAR-T治疗前3个月内	2（5.1）	2（22.2）	2（18.2）		
CAR-T治疗后3个月内	0（0）	1（11.1）	2（18.2）		
CAR-T治疗后3个月后	37（94.9）	6（66.7）	7（63.6）		
疗效评价[例（%）]				9.989	0.007
完全缓解/部分缓解	36（92.3）	6（66.7）	6（54.5）		
疾病稳定	0（0）	1（11.1）	1（9.1）		
疾病进展	3（7.7）	2（22.2）	4（36.4）		
新冠病毒疫苗接种[例（%）]				−2.158	0.031
未接种	32（82.1）	5（55.6）	6（54.5）		
接种至少1针	7（17.9）	4（44.4）	5（45.5）		
B细胞再生障碍[阳性例数/总例数（%）]	4/19（30.0）	3/5（60.0）	9/9（100.0）	−3.860	<0.001
中性粒细胞减低[阳性例数/总例数（%）]	3/10（30.0）	2/5（40.0）	4/10（40.0）	−0.456	0.648
淋巴细胞减低[阳性例数/总例数（%）]	5/10（50.0）	3/6（50.0）	7/10（70.0）	−0.888	0.375

注 CAR-T：嵌合抗原受体T细胞；R/R B-NHL：复发/难治性B细胞非霍奇金淋巴瘤；新冠病毒：新型冠状病毒。^a^包括滤泡性淋巴瘤、原发纵隔大B细胞淋巴瘤、套细胞淋巴瘤、灰区淋巴瘤

2. 免疫功能分析：对患者诊断新冠病毒感染时的免疫功能与感染严重程度的相关性进行评估。新冠病毒感染的中位时间为589（−88～1 819）d，其中6例（10.2％）患者在CAR-T细胞治疗前3个月内发生新冠病毒感染，3例（5.1％）在CAR-T细胞治疗后3个月内感染，50例（84.7％）在CAR-T细胞治疗后3个月后感染；新冠病毒感染多为CAR-T治疗结束3个月后，且轻型的比例更高，而CAR-T治疗前后3个月内若发生新冠病毒感染，中型与重型/危重型比例不低于轻型患者（*H*＝10.065，*P*＝0.007）。诊断新冠病毒感染时，48例（81.4％）患者处于疾病完全缓解或部分缓解状态，2例（3.4％）处于疾病稳定状态，9例（15.3％）处于疾病进展状态，完全缓解/部分缓解病例轻型感染更多见，而疾病进展患者重型比例更高（*H*＝9.989，*P*＝0.007）。在可获知B细胞状态的33例患者中，16例患者发生新冠病毒感染时处于BCA状态，处于BCA状态患者随疾病严重程度增加构成比增加，9例（100.0％）重型/危重型患者全部处于BCA状态。59例患者中仅16例（27.1％）既往接受至少1针新冠病毒疫苗接种，未接种疫苗患者轻型比例更高（*z*＝−2.158，*P*＝0.031）。中性粒细胞及淋巴细胞减低与否与新冠病毒感染严重程度差异无统计学意义（表1）。

3. 新冠病毒感染的临床特点和治疗情况：59例患者新冠病毒感染的中位持续时间为12（3～67）d，接受自体造血干细胞移植患者中位持续时间为17（4～66）d，接受单独CAR-T治疗患者的中位持续时间为10.5（3～67）d，差异无统计学意义（*z*＝−1.79，*P*＝0.073）。最高体温：23例（39.0％）患者≥38.5 °C，14例（23.7％）为37.3 °C～<38.5 °C，22例（37.3％）<37.3 °C。7例（11.9％）患者合并感染。抗病毒治疗：13例（22.0％）患者服用奈玛特韦/利托那韦，其中7例服用2个疗程或以上奈玛特韦/利托那韦；2例（3.4％）患者口服莫诺拉韦，4例（6.8％）患者口服阿兹夫定，10例（16.9％）患者输注了新冠病毒感染康复者恢复期血浆。抗炎治疗：11例（18.6％）患者在治疗期间使用了糖皮质激素，3例（5.1％）使用了托珠单抗，11例（18.6％）应用静脉注射免疫球蛋白。共17例（28.8％）患者因新冠病毒感染住院治疗，其中1例需要ICU监护治疗；10例（16.9％）需要呼吸支持治疗，主要包括鼻导管、面罩吸氧和机械通气。3例患者因淋巴瘤进展死亡。图1中列出了2例长期感染新冠病毒（例1为重型，新冠病毒感染持续66 d；例2新冠病毒感染持续67 d，危重型感染）和2例再感染患者（例3第三次新冠病毒阳性距离第二次阳性间隔107 d；例4第二次新冠病毒阳性距离首次阳性间隔117 d）的临床特点和治疗情况。4例患者都采用了2个疗程或以上奈玛特韦/利托那韦进行抗病毒治疗。8例患者进行了新冠病毒抗体检测，其中1例未输注过康复者血浆的患者，新冠病毒抗体阴性。按1∶2选取同一时间段、同一医院，年龄和性别匹配且进行该项检验的非血液肿瘤门诊患者为对照组，结果见图2。7例输注康复者血浆的患者有较低的抗新冠病毒抗体IgG水平［6.66±3.42）S/CO对（9.24±0.78）S/CO，*t*＝2.74，*P*＝0.013］。

**图1:**
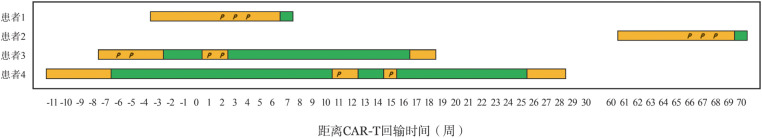
新型冠状病毒持续阳性和反复感染患者的抗病毒治疗（黄色为新冠检测阳性，绿色为新冠检测阴性，P指服用奈玛特韦/利托那韦）

**图2:**
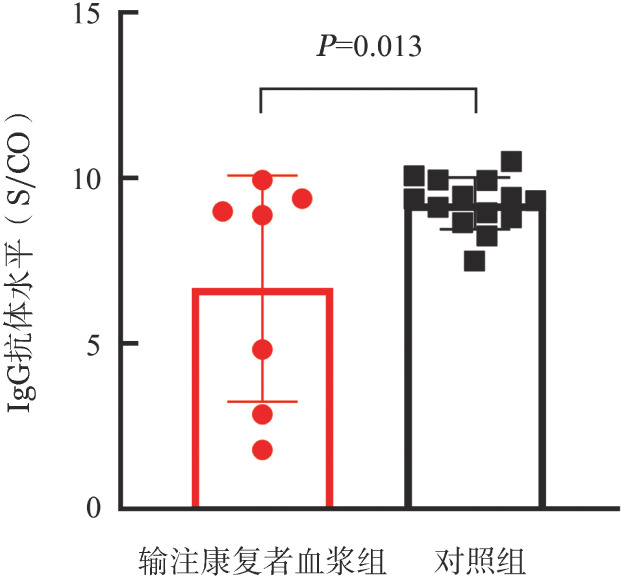
输注康复者血浆组（7例）和对照组（14例）的抗新型冠状病毒抗体IgG水平比较

4. 新冠病毒重症感染的影响因素分析：为了确定与新冠病毒重症感染相关的患者特征，结局事件设为重症感染（包括重型和危重型，共11例），自变量包括年龄、性别、是否接受过自体造血干细胞移植、是否接种新冠病毒疫苗、末次使用CD20单抗间隔时间、新冠病毒感染距离CAR-T回输的时间、新冠病毒感染时疾病状态、合并基础疾病、BCA。单因素分析显示，与新冠病毒重症感染显著相关的因素包括：年龄>55岁（*OR*＝5.32，95％*CI* 1.11～25.93，*P*＝0.045）、接受过自体造血干细胞移植（*OR*＝6.65，95％*CI* 1.50～22.63，*P*＝0.009）、疾病进展状态（*OR*＝5.94，95％ *CI* 1.48～30.20，*P*＝0.032）、BCA（*OR*＝20.0，95％ *CI* 2.20～181.56，*P*<0.001）（表2）。由于本研究样本量有限，且可评估BCA的病例数仅33例，未进一步行多因素分析。

**表2:** R/R B-NHL患者新型冠状病毒重症感染的单因素分析

因素	*OR*	*95%CI*	*P*值
年龄>55岁	5.32	1.11~25.93	0.045
男性	1.90	0.48~6.35	0.506
接受过自体造血干细胞移植	6.65	1.50~22.63	0.009
未接种疫苗	0.36	0.11~1.36	0.149
末次使用CD20单抗间隔时间<90 d	1.52	0.23~8.74	1.000
CAR-T治疗前后3个月内诊断新型冠状病毒感染	4.91	1.22~25.30	0.053
疾病进展状态	5.94	1.48~30.20	0.032
合并基础疾病	2.80	0.74~9.50	0.149
B细胞再生障碍^a^	20.0	2.20~181.56	<0.001

注 R/R B-NHL：复发/难治性B细胞非霍奇金淋巴瘤；^a^ 33例患者可获得CD19阳性淋巴细胞比例数据，由于B细胞再生障碍组均为新型冠状病毒感染重型和危重型，四格表中存在零计数，采用Haldane修正，即在每个单元格的计数中添加0.5，以避免在计算*OR*时出现除以零的错误

5. BCA病例特征：33例患者可获得CD19阳性淋巴细胞比例数据，其中BCA组16例，非BCA组17例。BCA与新冠病毒感染严重程度见表1、表2，进一步评估BCA组与非BCA组接受自体造血干细胞移植、新冠病毒感染持续时间、合并感染、抗病毒治疗以及是否住院治疗方面的差异。结果见表3，BCA组新冠病毒感染持续时间>14 d患者占比、抗病毒治疗及住院治疗患者占比明显高于非BCA组（均*P*<0.01），其他特征差异无统计学意义。

**表3:** B细胞再生障碍（BCA）与新型冠状病毒感染［例（％）］

特征	BCA组（16例）	非BCA组（17例）	*P*值^b^
接受自体造血干细胞移植	8（50.0）	3（17.6）	0.071
感染持续时间>14 d	12（75.0）	5（29.4）	0.015
合并感染	4（25.0）	2（11.8）	0.398
抗病毒治疗^a^			<0.001
2个疗程及以上	7（43.8）	0（0）	
1个疗程	4（25.0）	1（5.9）	
未治疗	5（31.2）	16（94.1）	
住院治疗	13（81.2）	1（5.9）	<0.001

注 ^a^抗病毒方案为奈玛特韦/利托那韦；^b^采用Fisher精确概率法计算

## 讨论

R/R B-NHL患者在诊断后通常经历多线治疗。接受CAR-T细胞治疗前的预处理，导致患者免疫功能缺陷。CD19单靶点、CD19/CD20双靶点或CD19/CD22双靶点CAR-T细胞定向消除恶性和健康的B细胞，会导致BCA和低丙种球蛋白血症[14]。对CAR-T细胞疗法反应不足的患者往往需要进一步治疗，包括PD-1抑制剂、BTK抑制剂，BCL-2抑制剂等，进一步加重了免疫抑制程度，更容易发生新冠病毒重症感染。

与健康人群相比，血液系统恶性肿瘤患者新冠病毒感染的重症率和病死率更高，因新冠病毒感染住院的血液系统患者死亡率约为34％[15]。一项2021年纳入56例患者的研究显示，接受CAR-T细胞治疗的血液系统恶性肿瘤患者新冠病毒感染住院率为80％，42.9％的患者需要氧气支持，病死率为41.4％[6]。本研究纳入的59例患者新冠病毒重症感染率、住院率和病死率明显低于上述报道，可能是因为本研究纳入时段主要流行奥密克戎变异株。相比德尔塔等其他变异株，国内外均有证据显示奥密克戎变异株肺部致病力明显减弱，临床表现已由肺炎为主衍变为以上呼吸道感染为主[16]。

在一项针对儿童和年轻成人患者接受CD19 CAR-T细胞治疗前后新冠病毒感染的研究中，在奥密克戎作为主要变异株出现后，23例患者中仅有1例（4.3％）因肺栓塞入院并进入ICU治疗[8]。本组患者新冠病毒重症感染率为18.6％，住院治疗率为28.8％，高于该研究。可能原因有：（1）本研究纳入患者的中位年龄为57（18～77）岁，高于该研究的16（1～27）岁，而年龄是发生新冠病毒重症感染的重要影响因素[6]；（2）本研究15.3％的患者发生新冠病毒感染处于疾病进展状态，而该研究95.7％的患者发生新冠病毒感染处于疾病缓解状态。

本研究显示，年龄>55岁、接受过自体造血干细胞移植、发生新冠病毒感染时处于疾病进展状态和BCA的患者更易发生新冠病毒重症感染。年龄更大和发生感染时处于疾病进展状态的患者更可能发生严重感染，这与针对血液系统恶性肿瘤和造血干细胞移植患者新冠病毒感染的报道[6],[17]–[18]结论类似。而接受过自体造血干细胞移植的患者免疫功能更差，导致更加严重的新冠病毒感染。BCA亦与更长的新冠病毒核酸阳性持续时间、更多的抗病毒治疗疗程及更高的住院率相关，这与接受造血干细胞移植和CAR-T细胞治疗患者的病毒携带时间延长[4]及感染奥密克戎变异株的免疫功能低下患者住院频繁，症状持续时间延长等报道[19]结论一致。本研究8例进行了新冠病毒抗体的检测者中1例未输注过康复者血浆的患者，新冠病毒抗体阴性，其余抗体滴度显著低于对照组，而且可能与输注康复者血浆有关，与既往造血干细胞移植和CAR-T细胞患者接受新冠疫苗的研究类似[20]–[21]，BCA患者体液免疫缺陷导致抗体形成障碍，这也提示我们，接受CAR-T治疗的患者（特别是处于BCA状态的患者）应当早期、及时、足量地进行抗病毒治疗及输注康复者血浆等治疗措施，预防重症感染，并根据病情严重程度给予免疫调节和支持治疗[22]；在新冠病毒感染康复后，更应注意防护，防止再感染。

本研究中，43例（72.9％）患者未接种新冠病毒疫苗，这些患者中轻型感染占比更高，单因素分析显示是否接种新冠病毒疫苗并非新冠病毒重症感染的影响因素。B细胞淋巴瘤患者对新冠病毒疫苗的免疫应答明显低于健康人群。但多数情况下，疫苗接种产生的免疫应答对患者具有保护效力。根据最新共识[23]，接受CAR-T细胞治疗的患者，应根据疾病状态和造血及免疫功能恢复情况，在CAR-T细胞回输3～6个月后接种新冠病毒疫苗。

除此之外，本研究中有6例患者在CAR-T细胞回输前发生新冠病毒感染，这些患者在感染时均为疾病进展状态。根据国内外专家意见[24]–[26]，在权衡推迟CAR-T细胞治疗的风险与基础疾病进展的风险后，该6例患者都不同程度地推迟了自体造血干细胞采集和（或）T细胞采集、预处理和CAR-T细胞治疗。

综上所述，本研究结果显示接受CAR-T细胞治疗的R/R B-NHL患者，尤其是年龄较大、接受过自体造血干细胞移植和处于疾病进展状态或BCA状态的患者，发生新冠病毒重症感染风险更高，持续时间更长。虽然奥密克戎变异株的致病力明显减弱，在拟行以及已经接受CAR-T细胞治疗患者中，新冠病毒感染仍然是亟须关注的问题。
